# Efficacy of Preoperative Exercise in Prehabilitation for Preventing Postoperative Sleep Disturbances and Pain: An Experimental Rat Model Study

**DOI:** 10.7759/cureus.79901

**Published:** 2025-03-01

**Authors:** Hirofumi Nakamoto, Moe Fujimoto, Megumi Nagata, Sekiyama Hiroshi, Shigehito Sawamura

**Affiliations:** 1 Department of Anesthesiology, Teikyo University School of Medicine, Tokyo, JPN

**Keywords:** electroencephalogram monitoring, postoperative pain, postoperative sleep disturbance, prehabilitation, preoperative exercise therapy

## Abstract

Background

Postoperative sleep disturbances and pain are common, negatively impacting recovery and quality of life. While various preventive strategies exist, the role of preoperative exercise in mitigating these effects remains underexplored.

Objective

This study evaluates the efficacy of preoperative exercise as a prehabilitation strategy to reduce postoperative sleep disturbances and pain in a rat model.

Methods

Male Wistar rats were divided into three groups: postoperative pain (PO) without preoperative exercise (N-group), PO with preoperative exercise (P-group), and a sham-operated control (S-group). Sleep patterns, including sleep duration and quality, were analyzed using EEG over a 72-hour period, starting at 8:00 a.m. on the first day of the experiment. Additionally, pain thresholds were assessed using the von Frey and Hargreaves tests.

Results

Compared to the N-group, the P-group exhibited reduced wake time and increased non-rapid eye movement (NREM) sleep duration. Additionally, the N-group showed increased wake time and decreased NREM sleep duration compared to the S-group, whereas no significant differences were observed between the P- and S-groups. The thermal allodynia test indicated a higher pain threshold in the P-group than in the N-group, although both remained lower than the S-group.

Conclusions

Our study demonstrates the efficacy of preoperative exercise as a nonpharmacological intervention for reducing postoperative sleep disturbances and alleviating pain. These findings highlight the potential benefits of prehabilitation for patients undergoing surgery.

## Introduction

Postoperatively, in response to surgical stress, patients frequently experience sleep disturbances, which are associated with various adverse events, including delirium, cognitive impairment, cardiovascular complications, heightened pain sensitivity, and impaired respiratory function [1,2]. Pain is often reported as a primary factor disrupting sleep after surgery [3,4], while sleep disturbances, in turn, have been shown to exacerbate pain [5,6]. Evidence suggests a bidirectional relationship between sleep disturbance and postoperative pain (PO), underscoring the need to explore interventions that address both simultaneously.

Preoperative exercise as part of prehabilitation has been reported to improve pain and physical function in the months following surgery [7,8]. However, to the best of our knowledge, its effects on postoperative sleep disturbances and pain remain unexamined. Therefore, this study aimed to investigate the efficacy of preoperative exercise in reducing postoperative sleep disturbances and pain using a rat model of PO. We hypothesized that preoperative exercise would directly mitigate these postoperative complications.

Sleep structures differ between humans and rodents. Humans sleep during the night (dark period) and follow a monophasic sleep pattern, whereas rodents sleep during the day (light period) and exhibit polyphasic sleep patterns. However, in both cases, the circadian rhythm maintains a certain periodicity and is closely related to sleep regulation. Given this, rodents serve as a suitable model for studying the effects of exercise on sleep. In humans, sleep disturbances can persist for three to four nights following surgery [5]. In PO model rats, pain from a plantar incision is most intense on the day of surgery and gradually subsides over the next few days [9]. Since postoperative sleep disturbances and pain are closely linked, we conducted a comprehensive analysis of sleep disturbances for three days after surgery.

## Materials and methods

Animals

The experimental protocols were approved by the Animal Care Committee of Teikyo University School of Medicine (Tokyo, Japan; approval no. 17-019) and conducted in accordance with the ARRIVE guidelines. In this study, five-week-old male Wistar rats (140-160 g) were purchased from the Hino Breeding Center (Tokyo, Japan) and housed under controlled conditions: a constant temperature of 24°C ± 0.5°C, relative humidity of 60% ± 2%, and a 12-hour light/dark cycle (lights on from 8:00 a.m. to 8:00 p.m.). Rats had ad libitum access to food and water. Measurements were taken on designated days, and at the conclusion of the experiment, all rats were euthanized using carbon dioxide.

PO models

One day before the experiment, seven-week-old male Wistar rats underwent a previously established PO modeling procedure [9]. Under anesthesia induced by an intraperitoneal injection of medetomidine, midazolam, and butorphanol (MMB; 0.375/2/2.5 mg/kg), a 1-cm vertical incision was made in the skin and fascia of the right plantar hindfoot, beginning 0.5 cm from the proximal heel and extending toward the toes. The plantaris muscle was elevated, stretched, and longitudinally incised. Hemostasis was achieved with pressure, and the skin was sutured.

Experimental groups

The rats were divided into three groups, each consisting of seven animals: PO rats without preoperative exercise (N-group), PO rats with preoperative exercise (P-group), and sham-operated control rats without preoperative exercise or surgery (S-group). Sleep patterns, including sleep duration and quality, were analyzed using EEG over a period of three days. Additionally, pain thresholds were assessed both preoperatively and at the conclusion of the experiment using the von Frey and Hargreaves tests [10,11].

Preoperative exercise paradigm

A running wheel (FWS-3002; 1.0-m circumference; Melquest Ltd., Toyama, Japan) was used as the preoperative exercise paradigm. The exercise protocol began when the rats were five weeks old, with the speed and intensity of forced running determined by measuring the velocity of the wheel’s revolution. The experimental regimen consisted of running at a speed of 10 m/min for 30 minutes per day, between 8:00 a.m. and 8:00 p.m., seven days a week, for two consecutive weeks before the start of the experiment. The speed and duration of the running sessions were selected based on a previously established protocol [12].

Electroencephalogram analysis

After establishing the PO model, the rat skulls were exposed, and EEG electrodes were attached following our previously established methods [13-15]. The electrodes were secured with dental cement, and each rat received five stainless-steel implants for epidural EEG recordings. The EEG screws were positioned on both sides of the prefrontal cortex (3.9 mm anterior to the bregma, ±2.0 mm lateral) and the occipital cortex (7.4 mm posterior to the bregma, ±5.0 mm lateral). A reference electrode was placed at the anterior position of the frontal bone (5.5 mm anterior to the bregma, 0.8 mm lateral to the right). Additionally, two steel wires were inserted into the muscles on the back of the head for EMG recordings. Data from the lead wires were collected through a socket fixed to the skull, and the left frontal-occipital lead data were analyzed. Following electrode implantation, the animals were allowed to recover from anesthesia.

The PO model induces thermal allodynia, mechanical hyperalgesia, and spontaneous pain behavior [16]. As a control, sham-operated rats underwent anesthesia with MMB and implantation of EEG and EMG electrodes, but no incision was made in the plantar hind foot [17].

EEG and EMG recordings were conducted continuously for 72 hours, from 8:00 a.m. on the first day of the experiment to 8:00 a.m. on the last day. On the day of the experiment, rats were first exposed to 5% isoflurane for initial anesthesia. Once immobilized, anesthesia was maintained with 2.5% isoflurane. The EEG-EMG recording cable was connected within a short period, after which the rats were placed in a cylindrical cage (inner diameter: 25 cm; height: 50 cm; volume: 25 L) for multichannel electrical recordings (Nihon Kohden Corporation, Tokyo, Japan). The room temperature was maintained at 24-25°C, with light and dark cycles beginning at 8:00 a.m. and 8:00 p.m., respectively. We compared the durations (in seconds) of wakefulness, rapid eye movement (REM) sleep, and non-REM sleep across groups.

Sleep stages were visually scored in 10-second epochs using Sleep Sign for Animals, version 3.2.0 (Kissei Comtech, Tokyo, Japan), based on fast Fourier transform analyses of EEG signals in the δ (0.75-4 Hz), θ (6-10 Hz), and α (8-13 Hz) frequency bands, along with parameters such as locomotion and EMG integral.

Wakefulness was identified by asynchronous low-amplitude, mixed-frequency EEG activity (>4 Hz) and high EMG activity (EMG integral >120 mV/s or locomotion >0). Non-REM sleep was characterized by an increase in EEG δ power above the threshold, along with reduced EMG activity (EMG integral <0.7 mV/s to 120 mV/s, EEG δ >173 mV² to 2133 mV², locomotion = 0). REM sleep was distinguished by an elevated EEG θ/(δ + θ) ratio and low EMG activity, meeting the threshold values (EEG θ/(δ + θ) ratio >0.5 to 0.73, locomotion = 0). Using this scoring system, changes in wakefulness, REM sleep, and non-REM sleep were analyzed over three days.

Pain threshold

Pain threshold was assessed using the von Frey and Hargreaves tests at two time points: before surgery (preoperation) and at the conclusion of the experiment on day 3.

Mechanical hypersensitivity was evaluated by determining the withdrawal threshold in response to mechanical stimulation of the right hind paw using von Frey filaments (0.008-300 g; DanMic Global, LLC, San Jose, CA, USA) [10]. Rats were individually placed in a metal mesh cage elevated 1 m above the ground and allowed to acclimate for 30 minutes. The tip of a von Frey filament was carefully applied to the incised plantar surface below the floor. Beginning with the lightest filament (0.008 g), each subsequent filament was applied until a withdrawal response was elicited. The test was performed three times, with a five-minute interval between trials. The average values obtained from the von Frey test are presented as a percentage of the control (% min).

Thermal allodynia was assessed by measuring the latency of hind paw withdrawal in response to radiant heat using a plantar test device (SN# 42012-400; IITC Life Science Inc., Woodland Hills, CA, USA), following a previously established procedure [10]. Each rat was placed in a sealed compartment on a glass surface, and an infrared beam was directed through the glass onto the hind paw, heating the skin to 39°C. To prevent tissue damage, a cutoff time of 20 seconds was set in cases where no withdrawal response was observed. The mean withdrawal latency for each measurement was calculated as the average of three trials, with five-minute intervals between them. The results of the Hargreaves test are expressed as the percentage of the maximum possible effect (%MPE), calculated using the following formula (cutoff time: 20 seconds):

\[ \%MPE = \left( \frac{\text{latency} - \text{baseline}}{\text{cutoff} - \text{baseline}} \right) \times 100 \]

Data analysis

The sample size (n = 7 rats per group) was determined using a previously established equation [18]. Statistical analyses were conducted using Excel Tokei (2012) (Social Survey Research Information Co., Ltd., Tokyo, Japan). Results are presented as mean ± SD. Data were analyzed using a one-way analysis of variance, followed by Bonferroni’s test, with statistical significance set at p < 0.05.

## Results

Electroencephalogram results

**Figure 1:**
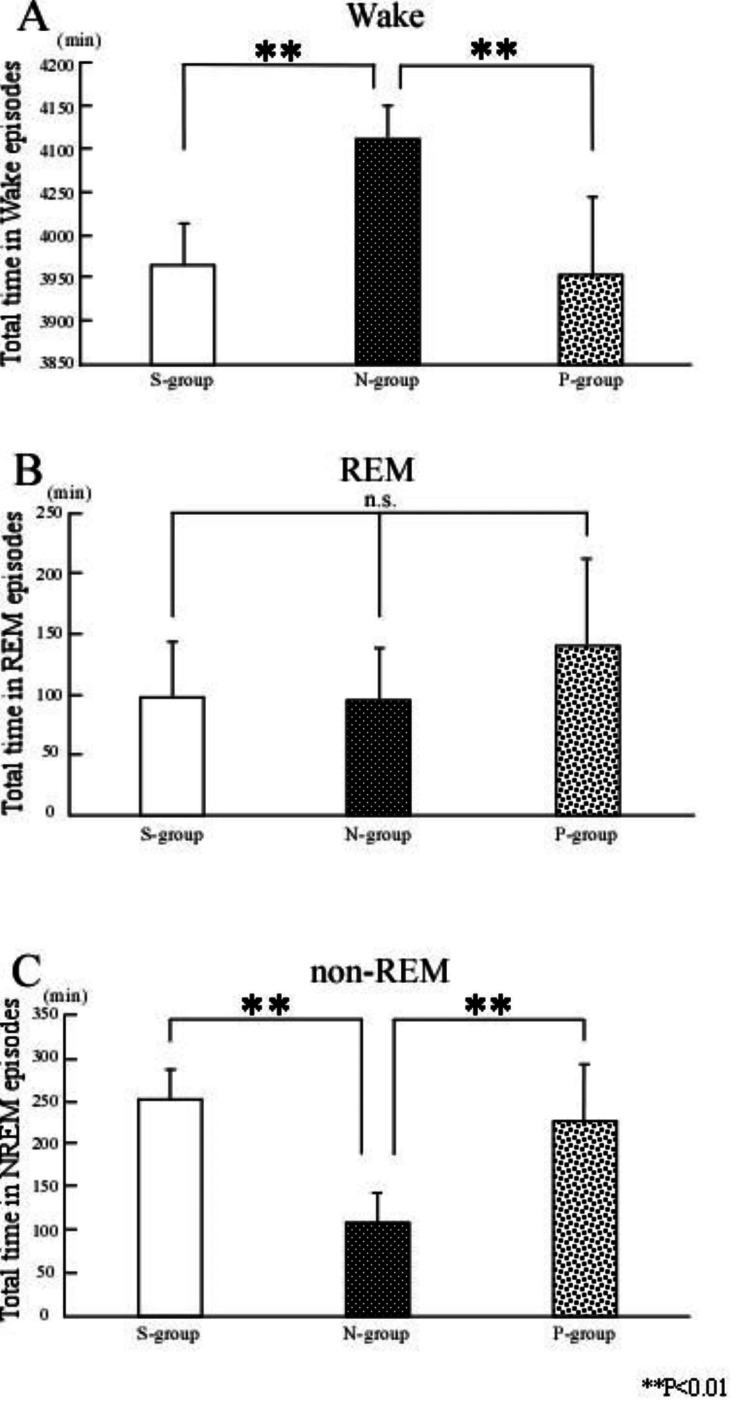
Effects of preoperative exercise on sleep disturbance The rats were divided into three groups: PO rats without preoperative exercise (N-group), PO rats with preoperative exercise (P-group), and sham-operated control rats (S-group). The durations of wake time, REM sleep, and non-REM sleep were compared across the three groups. Compared with the N-group, rats in the P-group exhibited a significant reduction in wake time and an increase in non-REM sleep time (p < 0.01), while no significant difference was observed in REM sleep time. Compared with the S-group, the N-group showed a significant increase in wake time and a decrease in non-REM sleep time (p < 0.01), with no significant difference in REM sleep time. Additionally, no significant differences were found between the P- and S-groups in wake time, non-REM sleep time, or REM sleep time. **p < 0.01; one-way analysis of variance (S-group, n = 7; N-group, n = 7; P-group, n = 7). Results are presented as mean ± SD. PO, postoperative pain; REM, rapid eye movement

Compared to the N-group (wake time: 4,114 ± 38.0 minutes; non-REM sleep time: 109 ± 32.55 minutes), the P-group exhibited a significant reduction in wake time and a significant increase in non-REM sleep time (wake time: 3,955 ± 91.23 minutes; non-REM sleep time: 224 ± 66.99 minutes; p < 0.001; Figure 1). However, no significant difference was observed in REM sleep time between the P- and N-groups (140 ± 72.15 minutes vs. 96 ± 41.86 minutes; ns).

Compared to the S-group (wake time: 3,968 ± 46.42 minutes; non-REM sleep time: 254 ± 31.7 minutes), the N-group showed a significant increase in wake time and a significant decrease in non-REM sleep time (wake time: 4,114 ± 38.0 minutes; p = 0.0012; non-REM sleep time: 109 ± 32.55 minutes; p < 0.001; Figure 1). No significant difference was found in REM sleep time between the N- and S-groups (96 ± 41.86 minutes vs. 99 ± 44.59 minutes; ns).

Additionally, there were no significant differences between the P- and S-groups in wake time (3,955 ± 91.23 minutes vs. 3,968 ± 46.42 minutes; ns), non-REM sleep time (224 ± 66.99 minutes vs. 254 ± 31.7 minutes; ns), or REM sleep time (140 ± 72.15 minutes vs. 99 ± 44.59 minutes; ns; Figure 1).

Pain threshold results

Preoperative pain thresholds did not differ among the S-, N-, and P-groups in either the thermal allodynia test (10.98 ± 9.70 vs. 9.33 ± 10.58 vs. -6.45 ± 22.86; ns) or the mechanical hyperalgesia test (94.28 ± 15.11 vs. 88.57 ± 19.51 vs. 103.8 ± 49.71; ns). Additionally, no visible differences were observed in the skin surface of the plantar hindfoot among the three groups.

**Figure 2:**
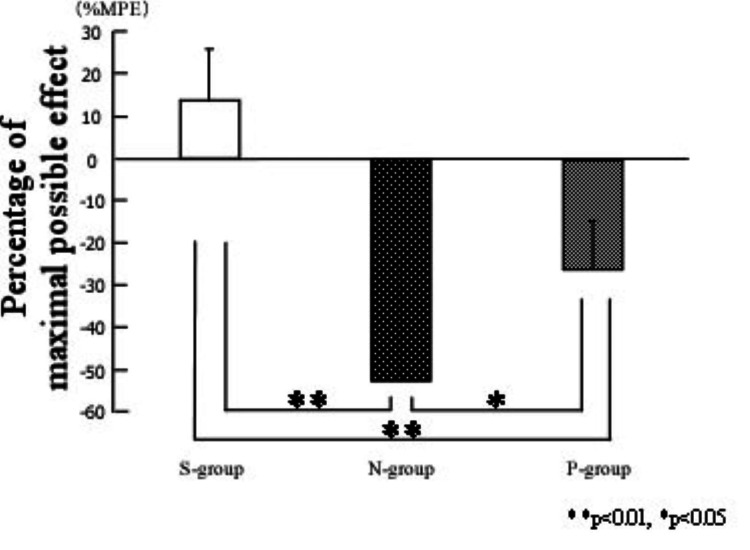
Effects of preoperative exercise on thermal allodynia In the thermal allodynia test at the end of the experiment, the pain threshold measured using the Hargreaves test was significantly lower in both the N-group and P-group compared with the S-group (p < 0.01). However, the pain threshold in the P-group was significantly higher than in the N-group (p < 0.05). **p < 0.01, *p < 0.05; one-way analysis of variance (S-group: n = 7, N-group: n = 7, P-group: n = 7). Results are presented as mean ± SD.

At the end of the experiment, the thermal allodynia test using the Hargreaves method showed a significant decrease in pain threshold in both the N-group (-53.15% ± 27.82%; p < 0.001) and P-group (-26.39% ± 11.32%; p = 0.0022) compared with the S-group (14.11% ± 11.72%; Figure 2). However, the pain threshold in the P-group was significantly higher than in the N-group (-26.39% ± 11.32% vs. -53.15% ± 27.82%; p = 0.045; Figure 2).

Similarly, in the mechanical hyperalgesia test using the von Frey method, the pain threshold at the end of the experiment was significantly lower in both the N-group (12.52% ± 9.55%; p < 0.001) and P-group (24.88% ± 4.63%; p = 0.0018) compared with the S-group (92.38% ± 38.38%). However, there was no significant difference in pain threshold between the N- and P-groups (12.52% ± 9.55% vs. 24.88% ± 4.63%; ns).

## Discussion

In this study, we investigated the effects of preoperative exercise on postoperative sleep disturbances and pain using a PO rat model. Postoperative sleep disturbances, characterized by increased wake time and decreased non-REM sleep time, were observed in our study. Patients undergoing surgery frequently experience significant sleep disturbances, including severe sleep deprivation, sleep fragmentation, and reductions in both slow-wave sleep (SWS) and REM sleep, as shown in polysomnographic studies [19]. Our findings demonstrated that preoperative exercise alleviated sleep disturbances in a PO rat model, aligning with previous reports [19]. This improvement was evidenced by decreased wake time and increased non-REM sleep time. Exercise has been shown to positively impact sleep by reducing sleep-onset latency and wakefulness after sleep onset while increasing SWS during non-REM sleep [4]. However, to our knowledge, no previous studies have examined the effects of preoperative exercise in prehabilitation on postoperative sleep outcomes.

Postoperative sleep disturbances may be linked to increased sympathetic activity, as heightened noradrenergic activity promotes wakefulness. Long-term exercise has been reported to attenuate the hypothalamic-pituitary-adrenal axis response, leading to reduced catecholamine secretion [20,21]. In our study, we hypothesize that preoperative exercise may improve postoperative sleep disturbances by reducing sympathetic activity via this pathway.

Additionally, we found that preoperative exercise mitigates thermal allodynia in a PO rat model. Exercise-induced hypoalgesia [22] refers to an increase in pain thresholds and a reduction in pain intensity in response to nociceptive stimuli during exercise. This effect is mediated by the release of endogenous analgesic compounds, including opioids, cannabinoids, serotonin, and noradrenaline, which modulate the endogenous pain inhibitory system [23,24].

Furthermore, myokines released during exercise activate the descending pain modulatory system in various brain regions [25]. Lactate, a key exercise metabolite, has been shown to inhibit pain responses via the transient receptor potential vanilloid 1 (TRPV1) signaling pathway [26]. Exercise has also been reported to increase pain thresholds by decreasing TRPV1 expression and reducing the excitability of nociceptive neurons in the dorsal root ganglia [27]. Based on these mechanisms, we speculate that the suppression of thermal allodynia observed in our study may involve the TRPV1 signaling pathway.

Previous studies have suggested a bidirectional relationship between sleep and pain, in which sleep disturbances exacerbate pain, which in turn worsens sleep quality [5]. Our results support the possibility that preoperative exercise not only reduces postoperative sleep disturbances but also alleviates PO, creating a beneficial cycle of improved sleep and pain relief. These findings suggest that preoperative exercise may be an effective strategy for minimizing postoperative sleep disturbances and pain.

However, this study has some limitations. Forced wheel running was conducted during the light cycle, a period when rodents are typically asleep and inactive. Exercise during this phase may influence physiological mediators with circadian rhythmicity [28]. Additionally, forced exercise in an unfamiliar environment could introduce physiological stress, potentially confounding the results [29]. In contrast, rodents naturally engage in voluntary running-wheel exercise at night [30]. Voluntary exercise may better preserve the circadian rhythm of laboratory rodents and reduce the stress associated with forced activity. Given its potential to minimize confounding factors, voluntary exercise could be a more suitable model for translating these findings to humans and warrants further investigation.

Exercise intensity is another critical factor. In human studies, maximal lactate steady state is commonly used to assess exercise intensity, and in rodents, blood lactate levels can be easily measured [31]. This approach helps determine appropriate exercise intensity and training protocols in animal models [31]. However, blood lactate levels were not measured in our study, making it difficult to precisely assess exercise intensity in the rats. This may partly explain why no significant differences in mechanical hyperalgesia were observed between the N- and P-groups.

Patients undergoing surgery often require integrated management of sleep disturbances and pain. PO relief typically involves multimodal analgesia, including opioid administration. In our PO rat model, no postoperative analgesics were used. The inclusion of analgesic treatment in future studies may offer additional clinical insights. Despite this methodological difference, our findings suggest that preoperative exercise could be a promising strategy to mitigate postoperative sleep disturbances and pain. In clinical practice, moderate walking may serve as an effective preoperative exercise intervention.

## Conclusions

We demonstrated that preoperative exercise effectively improves postoperative sleep disturbances while simultaneously alleviating pain. Our findings provide new insights into the benefits of incorporating preoperative physical activity into prehabilitation strategies to enhance patient recovery. Further research is needed to identify the optimal type and intensity of moderate preoperative exercise for these patients.
